# Supplementary material for: Modulation of the Bile Acid Enterohepatic Cycle by Intestinal Microbiota Alleviates Alcohol Liver Disease
Source: Cells. 2022 Mar 11;11(6):968. doi: 10.3390/cells11060968 (PMC8946080; doi:10.3390/cells11060968)
Supplement: Supplementary file 1 [file cells-11-00968-s001.zip › cells-1583268-supplementary.pdf]

Supplemental Table S1

**Oligos**	**5' Forward 3'**	**5' Reverse 3'**
ASBT	TGG-GTT-TCT-TCC-TGG-CTA-GAC-T	TGT-TCT-GCA-TTC-CAG-TTT-CCA-A
BSEP	GGA-TGG-TTT-GAC-TGC-ACT-TCT-G	AGA-GGA-CTG-ACA-GCG-AGA-ATC-A
Ccl2	AGG-TCC-CTG-TCA-TGC-TTC-TG	TCT-GGA-CCC-ATT-CCT-TCT-TG
Ccl3	Ref Qiagen : QT00248199	
CCR2	Ref Qiagen : QT02276813	
CD36	GTC-AAC-ATA-TTG-GTC-AAG-CCG-C	CCA-CTC-CAA-TCC-CAA-GTA-AGG-C
Cyp 7a1	Ref Qiagen : QT00121569	
Cyp 8b1	Ref Qiagen : QT00155778	
Cyp 27a1	Ref Qiagen : QT00155778	
Fabp1	GAT-TTC-TGA-CAC-CCC-CTT-GA	TGC-AGA-GCC-AGG-AGA-ACT-TT
Fatp4	CAC-GAA-TCA-GAA-CAG-AGA-GGC	TGC-TTT-GGT-TTC-TGG-GAC-TT
FGF15	GAG-GAC-CAA-AAC-GAA-CGA-AAT-T	ACG-TCC-TTG-ATG-GCA-ATC-G
FXR	CCA-ACC-TGG-GCT-TCT-ACC-C	CAC-ACA-GCT-CAT-CCC-CTT-T
Gapdh	GTG-GAC-CTC-ATG-GCC-TAC-AT	TGT-GAG-GGA-GAT-GCT-CAG-TG
Glut2	TCT-TCA-CGG-CTG-TCT-CTG-TG	AAT-CAT-CCC-GGT-TAG-GAA-CA
Glut5	AGA-GCA-ACG-ATG-GAG-GAA-AA	CCA-GAG-CAA-GGA-CCA-ATG-TC
IBABP	GAC-GGA-CAG-GAC-TTC-ACC-TG	TTG-CCA-CCC-TCC-ATC-TTC-AC
IL1b	AAG-GTC-CAC-GGG-AAA-GAC-AC	AGC-TTC-AGG-CAG-GCA-GTA-TC
MDR1	TAC-GAC-CCC-ATG-GCT-GGA-TC	GGT-AGC-GAG-TCG-ATG-AAC-TG
MRP2	GCT-TCC-CAT-GGT-GAT-CTC-TTC	ATC-ATC-GCT-TCC-CAG-GTA-CTG
MRP3	TAC-AGG-AAG-GCT-GTC-AT	GGA-TCT-GCC-AGA-GGA-AGT-AT
NOS2	CCA-AGC-CCT-CAC-CTA-CTT-CC	CTC-TGA-GGG-CTG-ACA-CAA-GG
OSTa	GTA-TTT-TCG-TGC-AGA-AGA-TGC-G	TTT-CTG-TTT-GCC-AGG-ATG-CTC
SGLT1	CGG-AAG-AAG-GCA-TCT-GAG-AA	AAT-CAG-CAC-GAG-GAT-GAA-CA
SHP	CGA-TCC-TCT-TCA-ACC-CAG-ATG	AGG-GCT-CCA-AGA-CTT-CAC-ACA
TGR5	GTC-AGC-TCC-CTG-TTC-TTT-GC	CAG-GAG-GCC-ATA-AAC-TTC-CA
TNF alpha	CAT-CTT-CTC-AAA-ATT-CGA-GTG-ACA-A	TGG-GAG-TAG-ACA-AGG-TAC-AAC-CC
UCP1	GCT-ACA-CGG-GGA-CCT-ACA-ATG	CGT-CAT-CTG-CCA-GTA-TTT-TGT-T

Table S1. Oligonucleotides used in this study.
